# Accommodating lithium into 3D current collectors with a submicron skeleton towards long-life lithium metal anodes

**DOI:** 10.1038/ncomms9058

**Published:** 2015-08-24

**Authors:** Chun-Peng Yang, Ya-Xia Yin, Shuai-Feng Zhang, Nian-Wu Li, Yu-Guo Guo

**Affiliations:** 1 CAS Key Laboratory of Molecular Nanostructure and Nanotechnology, and Beijing National Laboratory for Molecular Sciences, Institute of Chemistry, Chinese Academy of Sciences (CAS), Beijing 100190, China; 2 University of Chinese Academy of Sciences, Beijing 100049, China

## Abstract

Lithium metal is one of the most attractive anode materials for electrochemical energy storage. However, the growth of Li dendrites during electrochemical deposition, which leads to a low Coulombic efficiency and safety concerns, has long hindered the application of rechargeable Li-metal batteries. Here we show that a 3D current collector with a submicron skeleton and high electroactive surface area can significantly improve the electrochemical deposition behaviour of Li. Li anode is accommodated in the 3D structure without uncontrollable Li dendrites. With the growth of Li dendrites being effectively suppressed, the Li anode in the 3D current collector can run for 600 h without short circuit and exhibits low voltage hysteresis. The exceptional electrochemical performance of the Li-metal anode in the 3D current collector highlights the importance of rational design of current collectors and reveals a new avenue for developing Li anodes with a long lifespan.

## Abstract (web summary)

A major problem with the use of lithium metal as the battery anode is the undesired lithium dendrite formation during cycling. Here, the authors show that the problem can be mitigated with a carefully designed three-dimensional porous current collector.

## Graphical abstract

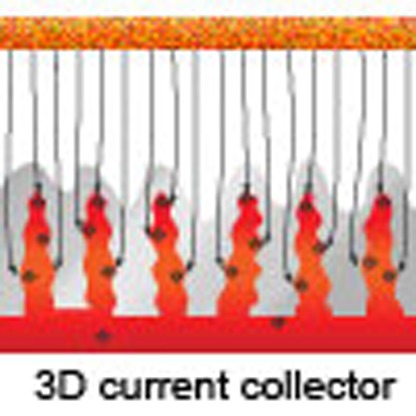

Electrochemical energy storage systems, for example batteries, with high-energy density and safe and clean features are critical for various applications such as smart electrical grid, electric vehicles and portable electronics. Advanced electrode materials are the key to high-energy batteries. Lithium metal is an ideal anode material in terms of energy density because it delivers an attractively high specific capacity (3,860 mA h g^−1^) and has the lowest reduction potential (–3.04 V versus standard hydrogen electrode)^1^. However, the use of Li metal as anode faces several hurdles. The most challenging one is the formation of Li dendrites during cycling, which causes safety hazards and exposes the Li-metal batteries to wide safety concerns^2^. The use of Li-ion batteries, which use rocking Li ions and Li-intercalating materials (such as graphite) instead of Li metal as anode, has successfully circumvented this problem and gained great success^3^,^4^,^5^. Nevertheless, with the energy density of Li-ion batteries approaching the theoretical value, they will soon no longer meet the demands for advanced energy storage. We are coming to an age beyond Li-ion batteries, in which advanced energy storage systems are necessary^6^.

In this context, Li-metal batteries, which used to be considered ‘unsafe', should be examined further. Advanced Li-metal batteries including Li–air (Li–O_2_), Li–S and Li–Se batteries have emerged as required because they can provide efficient energy storage^7^,^8^,^9^,^10^,^11^. Considerable efforts have been devoted to develop these novel Li-metal batteries. The electrochemical performances of the air, S and Se cathodes have been significantly improved^12^,^13^,^14^,^15^,^16^,^17^,^18^,^19^,^20^. The issues of Li anode, however, remain unsolved. The persistent challenge is the formation of dendritic Li during Li plating, which would lead to low Coulombic efficiency, short cycle life, internal short circuits and even catastrophic cell failure^21^,^22^. Only when the Li anode is improved markedly we can build viable Li-metal batteries for energy storage applications. Recently, attempts have been made to tackle the problems of Li anode^23^. Studies have been concentrated on the liquid electrolytes with optimal solvents and Li salts for stable interfaces between the Li metal and electrolytes^24^,^25^,^26^. Various electrolyte additives, such as Cs^+^ and Rb^+^ ions^27^, LiF (ref. 28), Cu(CH_3_COO)_2_ (ref. 29), have been explored to restrain dendrite formation and reinforce protection for the Li surface. In addition, it is found effective to suppress the Li dendrites using physical protective layers such as a carbon nanosphere layer^30^, BN/graphene^31^, a graphite layer^32^ and other *ex situ* coated protective layers^33^. As for the Li metal itself, several studies have indicated that Li alloys such as Li–Al (ref. 34) and Li–B alloys^35^,^36^ can change Li deposition behaviour. Although these studies have contributed to improve Li-metal anodes, the dendrite-forming deposition nature of Li metal has hardly been changed. Thus, new means to suppress dendrite formation will provide additional approaches to improve the performance of Li-metal anode.

As a key component of the anode, the current collector could also have a significant influence on the Li anode. The current collector affects the nucleation at the initial period of Li plating, which is decisive for the morphology of the subsequently plated Li. However, the role of the current collector for the Li-metal anode has not been investigated thoroughly. Most of the current collectors used in the Li batteries are planar, such as conventional Cu and Li foils. The initial plating of Li on planar current collector is prone to inhomogeneous Li particle deposition, followed by the growth of Li dendrites on the Li particles. In this study, we show that a three-dimensional (3D) current collector with a submicron-sized skeleton and porous structure can change the plating behaviour. When the porous Cu foil is used as the 3D current collector, Li grows on the submicron-sized Cu skeleton and fills the pores of the 3D current collector. With Li-metal anode accommodated in the 3D current collector, we are able to get Li metal anodes free from the Li dendrite crux and remarkably improve the lifespan of Li-metal anodes.

## Results

### Preparation for the 3D Cu foil with submicron skeleton

**Figure 1:**
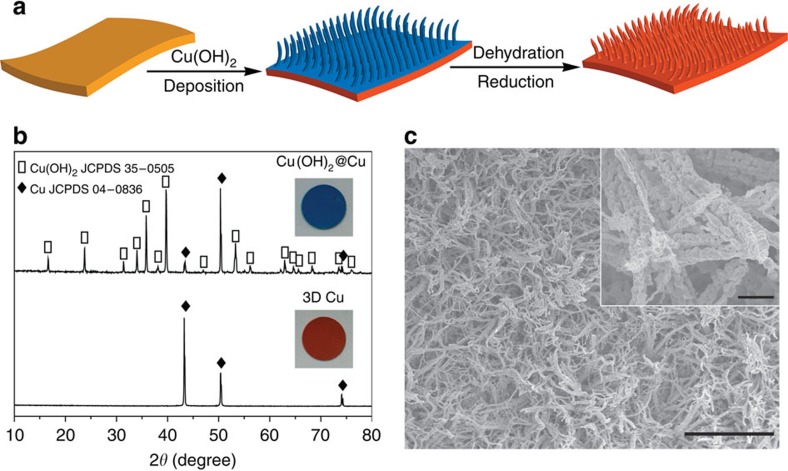
Preparation and characterization for 3D Cu foil. (**a**) Schematic presentation of the procedures to prepare a 3D porous Cu foil from a planar Cu foil. (**b**) X-ray diffraction profiles of Cu(OH)_2_ on the Cu foil and the final 3D Cu foil. The insets show digital images of the corresponding samples. (**c**) SEM images of the porous Cu (scale bar, 50 μm). The inset shows the high-magnification image (scale bar, 2 μm).

The 3D porous Cu foil is fabricated from a commercial planar Cu foil via a facial and scalable method. The preparation process for the 3D porous Cu foil is schematically presented in Fig. 1a. The planar Cu foil was first immersed in an ammonia solution to allow Cu(OH)_2_ deposition by self-assembly. Major chemical reactions took place during this period are (refs 37, 38)


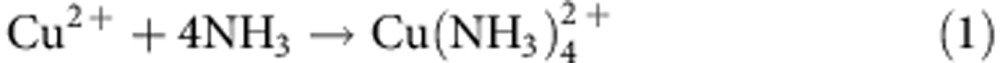



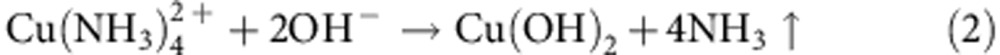


The Cu(OH)_2_ on the Cu foil was dehydrated to get CuO, which used to be applied as anodes, supercapacitors and others^38^,^39^. It was further reduced to get the porous Cu as the porous current collector. From the powder X-ray diffraction profiles (Fig. 1b), it is evident that the cyan layer on the Cu foil is Cu(OH)_2_ and it is completely converted into Cu after dehydration and reduction (see Supplementary Fig. 1 for the photographs of the samples). The final Cu foil shows a 3D structure composed of bundles of Cu fibres, as shown in the scanning electron microscopy (SEM) images (Fig. 1c) The Cu fibres are several submicron in diameter and have a nanosized protuberant secondary structure on the surface, as shown in the inset in Fig. 1c. The Cu submicron fibres are roughly perpendicular to the foil, forming a jungle-like porous layer (see side view image in Supplementary Fig. 2). According to mercury porosimetry analysis (Supplementary Fig. 3), the median pore diameter of the 3D Cu is 2.1 μm. The 3D Cu foil has an areal pore volume (pores <5 μm) of 1.5 × 10^−3^ cm^3^ cm^−2^ and a high areal pore area of 45 cm^2^ cm^−2^ (pore area per unit geometric area).

### Li-metal deposition behaviour

**Figure 2:**
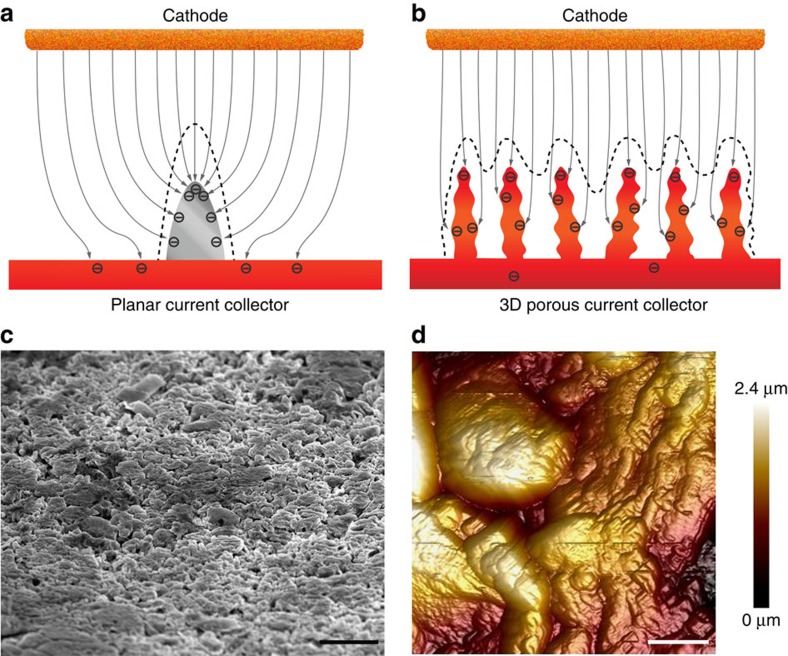
Electrochemical deposition behaviours of Li-metal anodes. Illustration of the proposed electrochemical deposition processes of Li metal on (**a**) planar current collector and (**b**) 3D current collector. The distribution of the electrons in the current collectors in the electrical field is schematically presented; the dashed lines illustrate the possible position where Li would be deposited. (**c**) Side view SEM image and (**d**) AFM height image of 2 mA h cm^−2^ of Li deposited on the 3D Cu foil with a submicron skeleton. Scale bars, 10 μm (**c**), 1 μm (**d**).

The 3D Cu foil was utilized to investigate the plating behaviour of Li metal on a 3D current collector. The pristine Cu foil was also tested as an example of planar current collectors. On a planar current collector, Li is apt to firstly form small Li dendrites (0.1–0.5 μm in diameter) on the smooth surface at the nucleation step. This nucleation mechanism has been well known and can be observed from the atomic force microscopy (AFM) image (Supplementary Fig. 4) at the nucleation step. The previously deposited small Li dendrite functions as a charge centre as the charges accumulate at sharp ends in the electric field (Fig. 2a). The subsequent Li metal is then deposited on these sharp ends and amplify the growth of the Li dendrites. In contrast, on the submicron skeleton of the 3D Cu foil, numerous protuberant tips on the submicron fibres function as the charge centres and nucleation sites. The electric field is roughly uniform and the charges are fairly homogeneously dispersed along the Cu skeleton. Therefore, Li is expected to nucleate and grow on the submicron Cu fibres with nanosized lumps, fill the pores of the 3D current collector, and eventually form a relatively even Li surface (Fig. 2b). To confirm this hypothesis, we plated Li on the planar and 3D Cu foils and disassembled the cells to observe the morphologies of the anodes. To focus on the effect of current collectors, we used 1 M lithium bis(trifluoromethane)sulfonimide (LiTFSI) dissolved in 1,3-dioxolane/1,2-dimethoxyethane (DOL/DME, 1:1 by volume) without any additives as electrolyte. Before further electrochemical procedures, the current collectors were first initialized by cycling at 0–1 V (versus Li^+^/Li) at 50 μA for five cycles to remove surface contaminations and stabilize the interface (Supplementary Fig. 5)^30^. A large area of mossy Li is observed on the planar Cu foil after depositing 2 mA h cm^−2^ of Li. A number of bumpy Li are found from the 52° side view image of the Li anode plated on the planar Cu. Li is deposited on the previously deposited Li dendrites and expands to larger and higher dendrites regardless of some area of bare Cu (Supplementary Fig. 6). The vertically grown dendrites can pierce through the separators and cause catastrophic cell failure. This plating behaviour of dendritic Li is consistent with previous reports and the drawbacks are well known^40^. For the 3D current collector, on the contrary, a relatively flat Li surface is obtained on the 3D Cu foil after depositing 2 mA h cm^−2^ of Li metal (Fig. 2c). According to the AFM image of the Li metal on the 3D current collector, the plated Li metal displays an undulating topography with gentle slopes whose height difference is <2.5 μm (Fig. 2d). No raised Li dendrites are found from the SEM and AFM images, thereby indicating that the possibility of the Li metal short-circuiting the cell is negligible. The morphology of the Li metal on the 3D Cu foil differs significantly from that on the planar Cu. The surface of the latter is so uneven that its height difference is beyond the measuring capability of AFM. The morphology of the Li-metal plated on the 3D current collector is in good agreement with the expectation, as illustrated in Fig. 2b. Therefore, by accommodating the Li metal into the 3D current collector, the growth of dendritic Li is effectively suppressed.

**Figure 3:**
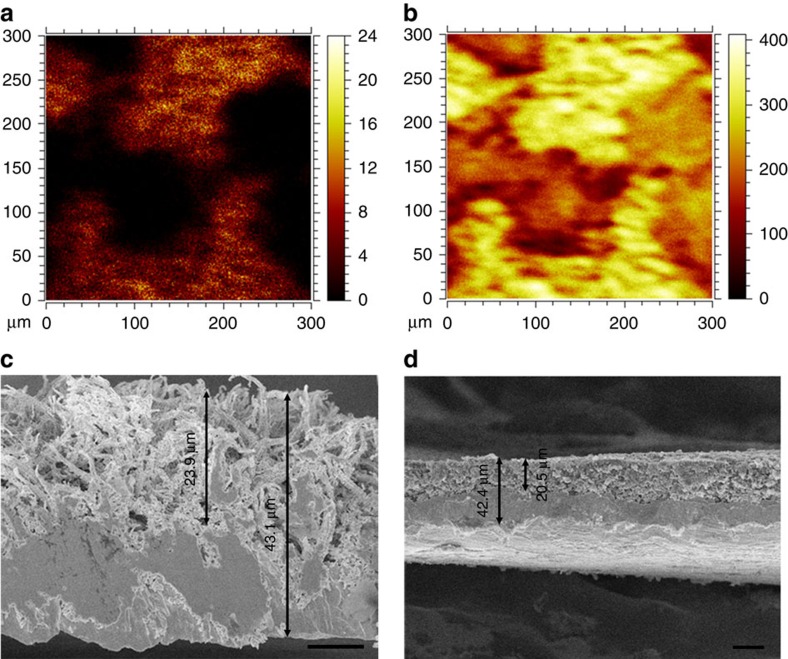
Spatial distribution of Li anode in 3D current collector. Elemental distribution images of (**a**) Cu^+^ and (**b**) Li^+^ of the Li-metal anode (2 mA h cm^−2^) deposited in the submicron-structured 3D Cu probed via ToF-SIMS. Cross-sectional view SEM images of (**c**) the pristine 3D porous Cu foil and (**d**) 2 mA h cm^−2^ of Li deposited on 3D porous Cu. Scale bars, 10 μm (**c**), 20 μm (**d**).

To further examine spatial distribution of the Li metal deposited in the 3D Cu current collector, we employed time-of-flight secondary ion mass spectrometry (ToF-SIMS) to probe the elemental distribution of Cu and Li in the Li-metal anode (2 mA h cm^−2^) in the 3D porous Cu current collector. The image of Cu^+^ (Fig. 3a) shows the Cu framework with pores. Li is plated mainly on the Cu skeleton and fills the pores of the 3D Cu foil (Fig. 3b). In addition, based on the depth profiles of Cu^+^ and Li^+^ (Supplementary Fig. 7) from the anode, their intensities with the sputter time (that is, depth distribution of Cu and Li) vary coincidently. These results confirm that the Li metal is accommodated into the 3D current collector rather than merely on the surface. The larger scale cross-sectional SEM images further demonstrate the accommodation of Li inside the 3D Cu. The thickness of the 3D Cu foil is ∼43 μm and that of porous layer is ∼24 μm (Fig. 3c). After plating 2 mA h cm^−2^ of Li metal, the porous layer is filled with Li, which is ∼20 μm in thickness (Fig. 3d). In addition, it is evident that Li metal is constrained in the 3D Cu foil in a fairly compact manner. The densely deposited Li anode contributes to high areal capacity. The elemental distribution images obtained via ToF-SIMS and the cross-sectional SEM images demonstrate that the Li metal is grown along the submicron Cu skeleton and is accommodated in the reserved porous structure. Even at higher current densities (2 and 5 mA cm^−2^), Li is deposited inside the 3D structure (Supplementary Fig. 8). The accommodation of Li could be attributed to the unique structure of the Cu submicron fibres, which provide a high surface area inside the 3D structure (45 times that of the geometric area, see Supplementary Fig. 3). The high surface area of 3D current collector provides more electroactive surface area for Li ions and electrons in the porous layer, where Li ions get electrons and deposit inside the 3D current collector. If the 3D current collector can provide sufficient pore volume for Li anode, the plating and stripping of Li can be controlled and the Li-metal anode will not form fatal dendrites that may pierce the separator.

**Figure 4:**
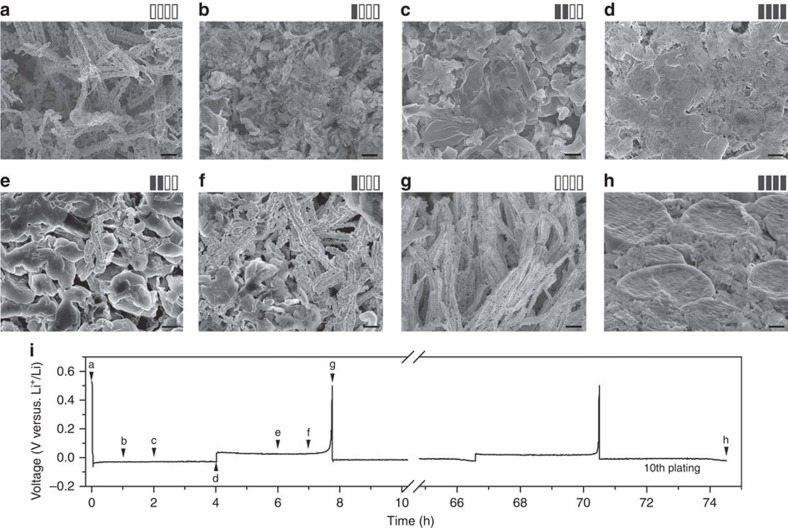
Morphology of Li-metal anode during plating/stripping. Top view SEM images of (**a**) pristine 3D porous Cu foil without Li metal and after plating (**b**) 0.5 mA h cm^−2^, (**c**) 1 mA h cm^−2^ and (**d**) 2 mA h cm^−2^ of Li metal into 3D current collectors; anodes after stripping (**e**) 1 mA h cm^−2^, (**f**) 1.5 mA h cm^−2^ and (**g**) 2 mA h cm^−2^ (that is, recharged to 0.5 V) from the Li anodes (2 mA h cm^−2^) with 3D current collectors. (**h**) Side view SEM image of the Li anode with the 3D current collector after 10 cycles. Scale bars, 2 μm. The rectangle symbols exhibit the amount of Li metal in each image; each solid rectangle represents 0.5 mA h cm^−2^ of Li. The Li plating/stripping states (**a**–**h**) are indicated in (**i**) galvanostatic discharge/charge voltage profile at 0.5 mA cm^−2^. Note that due to the nature of the *ex situ* method, the profile is only an indication for the Li plating/stripping states in **a**–**h**, and is not necessarily the real test result of each sample.

We also investigated the growing and stripping process of Li in detail to find the morphology evolution of the Li anode and the structural stability of the substrate (Fig. 4). From the SEM images of anodes with increasing Li amounts on the 3D Cu foil (Fig. 4a–d), Li grows on the Cu skeleton and gradually fills the pores of the porous Cu foil, forming an even surface (Fig. 4d). Given that the initially nucleated Li particles are several submicron in diameter, as reported in previous literature^40^ and demonstrated by the AFM image (Supplementary Fig. 4), the submicron Cu skeleton with nanosized protuberances is particularly appropriate for the nucleation and growth of Li metal. The Li metal can also be stripped reversibly from the submicron-structured 3D Cu current collector. As shown in the SEM images of the Li anodes during stripping (Fig. 4e–g), the Li metal is gradually stripped from the current collector and is completely stripped after recharging to 0.5 V. Furthermore, the submicron Cu fibres remain structurally stable after Li stripping (Fig. 4g). After repeated cycles, the surface of the Li-metal anode still keeps even without presence of protruding Li dendrites (Fig. 4h and Supplementary Fig. 9). The dendritic problem is noticeably mitigated as the Li metal can plate and strip reversibly forming an even surface. We characterized the solid electrolyte interphase (SEI) film on the Li-metal anode with the 3D current collector after 10 cycles by X-ray photoelectron spectroscopy (XPS). According to the XPS spectra (Supplementary Fig. 10), the SEI film on the 3D Cu current collector is composed of ROLi, ROCOOLi, LiF and so on, in agreement with that reported in literature using a similar electrolyte^41^. The SEI film is generally stabilized during the initial cycling, which was applied to facilitate the interface stabilization^30^. After initialization, as indicated in Supplementary Fig. 5, capacity contributed from the SEI formation is negligible compared with that from Li metal. We also note that despite the fibrous structure, the Cu submicron fibres themselves do not penetrate the separator because they are obtuse and flexible. This result is demonstrated by the electrochemical performance of the Li-metal anodes with the 3D porous Cu current collectors and Celgard separators in Fig. 4i and in the following discussion.

### Electrochemical performance

**Figure 5:**
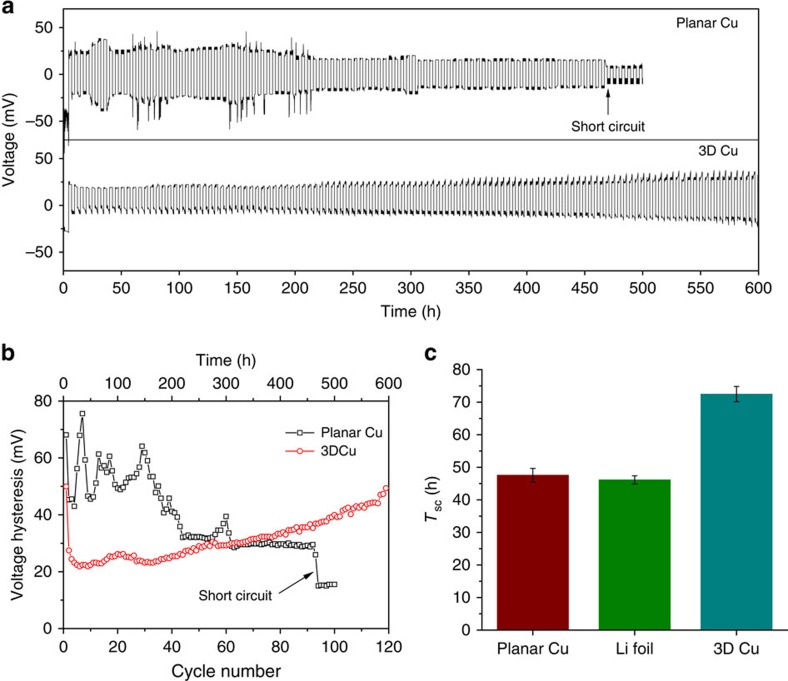
Electrochemical performance of Li metal anodes. (**a**) Voltage profiles and (**b**) average voltage hysteresis of Li metal plating/stripping at 0.2 mA cm^−2^ in symmetric Li|Li@Cu cells with planar or 3D Cu foil as current collector. (**c**) Average short-circuit time *T*_sc_ for unidirectional galvanostatic Li plating from Li foil to planar Cu foils, Li foils and 3D Cu foils in symmetric cells at 0.5 mA cm^−2^. The error bars are standard deviations obtained from at least three independent cells for each current collector.

We examined the electrochemical behaviour of Li plating/stripping and the cycling stability on different current collectors by comparing the galvanostatic discharge/charge voltage profiles of the Li electrode with the planar or 3D porous Cu current collectors (Li@Cu) in symmetric Li|Li@Cu cells (Fig. 5a). The symmetric cell contained a Li counter/reference electrode and a Li@Cu working electrode. A hollow spacer was used substituting for the Celgard separator to allow possible internal short circuits (see Methods and Supplementary Fig. 11 for more details). During Li plating/stripping at 0.2 mA cm^−2^ (except the initial plating at 0.5 mA cm^−2^), the Li electrode on the planar Cu shows random voltage oscillations, which could possibly be caused by the unstable Li/electrolyte interface and electrical disconnection because of repeated growth/corrosion of dendritic Li (ref. 30). After cycling for ∼470 h, the continuously growing Li dendrites on the Cu foil finally reach the counter electrode and short-circuit the cell. In contrast, Li plating/stripping on the 3D Cu foil exhibits exceptional cycling stability with negligible potential fluctuation. After cycling for 600 h, no sign of short circuit is observed, indicating that the growth of dendritic Li has been significantly retarded. The short circuit phenomenon and voltage variation can be observed clearly from the voltage hysteresis curves (Fig. 5b). The voltage hysteresis is the difference between the voltages of Li stripping and plating and is mainly determined by the current density, interfacial properties and charge transfer resistance^30^,^31^. At a current density of 0.2 mA cm^−2^, Li plating/stripping on the planar Cu foil shows an irregular fluctuating voltage hysteresis because of the unstable interface of Li on the planar Cu. The voltage hysteresis drops to ∼10 mV abruptly after 94 cycles (that is, 470 h) because of the dendrite-induced short circuit. The voltage hysteresis of Li plating/stripping on the 3D Cu foil, however, is generally stable without any irregular oscillations. Although the voltage hysteresis on 3D Cu foil increases gradually after the initial cycle, it is still <50 mV after cycling for 600 h, which is close to or better than the previously reported results^30^,^31^. This result could be attributed to the larger surface area of the porous Cu than that of the planar one. The larger electroactive area can provide a larger Li/electrolyte interface, lower the practical current density, and reduce the charge transfer resistance during cycling compared with the planar Cu (Supplementary Fig. 12). The reduced hysteresis of the Li anode is in favour of low voltage polarization during discharge/charge in full Li-metal batteries.

Unidirectional galvanostatic plating of Li was applied to accelerate the short-circuit analysis. Li was continuously plated onto different current collectors, including planar Cu foil, Li foil and 3D porous Cu foil, at 0.5 mA cm^−2^ until short circuit. The statistical results of the short-circuit time (*T*_sc_, Fig. 5c) prove that dendritic Li growth on 3D Cu foil is considerably slower than on the planar foils, and the cell life of the Li anodes with porous current collectors is much longer. In fact, as long as there is room for Li accommodation in the 3D current collector, Li plating will be restricted within the reserved pores and will not cause cell failure. Therefore, a long lifespan of the Li-metal anode is expected with the 3D current collector.

The Coulombic efficiencies of the Li anodes on the planar Cu and 3D Cu are compared in Supplementary Fig. 13. On the planar Cu, the plating/stripping efficiency of Li metal changes from 70% to over 100% because of the unstable morphology of the Li dendrites and the anode/electrolyte interface during cycling. Because of the submicron structure in the porous Cu foil, the efficiency of Li on the 3D Cu is ∼97% after 50 cycles at 0.5 mA cm^−2^, which is considerably more stable than that on the planar Cu foil. The initial Coulombic efficiency of the Li anode with 3D current collector is 71% in DOL/DME. To further improve the initial Coulombic efficiency of the Li anode, LiNO_3_ and lithium polysulphide are used as additives in the electrolyte, which have been reported to play a synergetic effect on Li anode^42^. The initial Coulombic efficiency can be remarkably improved to 93% by adding 1% LiNO_3_ and 0.005 M Li_2_S_6_ in the electrolyte and it is finally stabilized to 98.5% (Supplementary Fig. 14).

Following the methods in literature^30^,^43^, the Li anode with 3D Cu current collector was assembled into a full cell against a LiFePO_4_ cathode. As shown in Supplementary Fig. 15a, the full cell shows a high capacity and cycling stability. As the preparation for the 3D Cu foils is facile and scalable (see a 3D Cu foil of ∼70 cm^2^ in Supplementary Fig. 1), large Li-metal anodes using the 3D Cu current collectors was prepared and assembled into a pouch cell. The cell is demonstrated feasible by powering an LED device (Supplementary Fig. 15b), indicating the potential of the 3D Cu current collector for practical application.

## Discussion

The pore volume, pore size and surface area are important parameters for 3D current collectors. For 3D current collectors, the pore volume of the porous structure determines the amount of Li that can be accommodated, that is, the areal capacity density of the anode. The porous layer of the 3D foil is ∼24 μm (Fig. 3c). According to the mercury porosimetry analysis, the volume of effective pores is 1.5 × 10^−3^ cm^3^ cm^−2^ (Supplementary Fig. 3). The pore volume can accommodate ∼0.8 mg cm^−2^ of Li metal. The areal capacity density of Li anode accommodated in the porous Cu foil is estimated to be up to ∼3.1 mA h cm^−2^. The areal capacity density can fulfil most of the present demands and can be improved simply by increasing the pore volume of the 3D Cu foil. In fact, by increasing the immersion time of the Cu foil in the ammonia solution, the porous layer of the 3D Cu foil can be increased to ∼40 μm with more abundant pores for Li accommodation (Supplementary Fig. 16).

In addition to the submicron-structured 3D Cu foil, there are other possible candidates for the 3D current collector. For example, fibrous Li_7_B_6_ derived from Li–B alloy has been reported as a 3D matrix for Li anode^35^,^36^. However, the 3D structure of the alloy is far less abundant than the porous Cu foil. Thus, the alloy is less satisfactory in terms of suppressing dendrite growth by the 3D structure, let alone its drawbacks of cost and mechanical strength compared with the porous Cu foil. Recently, nanostructured graphene framework was also found helpful for stable and efficient Li deposition^44^. The reported graphene network has hierarchical pores with an average pore size of 10 nm. Another possible candidate as 3D current collector is Cu foam, which is commercially accessible and provides a large pore volume for Li. However, the average pore size of the Cu foam is too large (170 μm) with a wide pore size distribution (Supplementary Fig. 17). With such a large pore size approaching macroscopic scale, the Cu foam is more of a conventional current collector without any of the effects of space constraint and dendrite suppression. Unlike the Li anode inside the 3D Cu foil with a submicron skeleton, the Li metal can be detached easily from the backbones of the Cu foam, thereby resulting in electric disconnection. From the digital and SEM images of the Li anode with the Cu foam current collector after 20th Li stripping, a large amount of ‘dead Li' is found in the intervals of the Cu backbones and even on the separator (Supplementary Fig. 18), resulting in a very poor plating/stripping efficiency. The plating/stripping processes of Li in the Cu foam are irreversible with an efficiency of only ∼40% (Supplementary Fig. 19). Therefore, the Cu foam with an inappropriately large pore size is not suitable as 3D current collector for the Li anode. In contrast, Li anode in the 3D current collector with a median pore size of 2.1 μm can be reversibly plated/stripped from the substrate (Fig. 4) with a high Coulombic efficiency. Therefore, the submicron structure of the 3D Cu current collector, on one hand, provides a high pore volume to accommodate the Li anode with a favourable capacity density, and on the other hand, possesses suitable submicron 3D structure for stable and reversible Li plating/stripping. These results highlight the importance of the pore size of 3D current collectors for Li-metal anodes.

**Figure 6:**
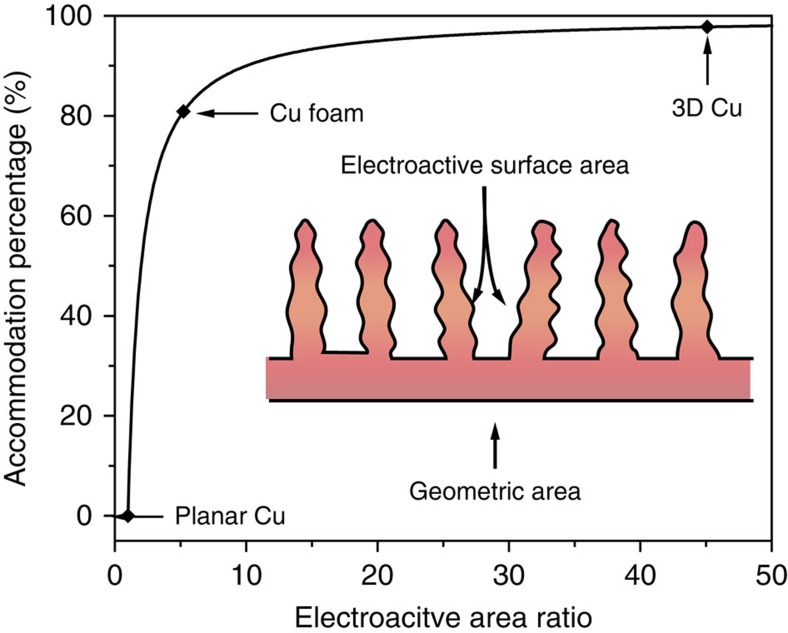
Li accommodation percentage with electroactive area ratio. The plot shows *η* as a simplified function of *r*. The electroactive surface area and geometric area of the current collector are illustrated in the inset. The planar Cu, Cu foam and 3D Cu are indicated in the plot.

As a most important parameter affecting Li plating, the electroactive surface area of a 3D current collector (the surface area exposed to the electrolyte, that is, total pore area here) is crucial for a sufficient electric contact between the Li anode and the substrate. The percentage of Li metal deposited inside the 3D structure (*η*) is generally determined by the ratio of electroactive surface area to geometric area of electrode (electroactive area ratio, *r*). Assuming that the electrons distribute uniformly on the skeleton and the current density is not too high (that is, Li-ion diffusion is not limited), Li will be deposited on the electroactive surface equiprobably. In this case, as depicted in Fig. 6, *η* is determined by *r*, according to the following formula


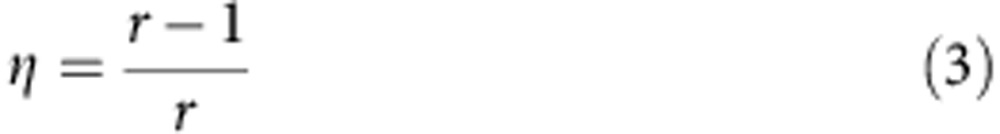


The textural parameters of planar Cu, Cu foam and 3D Cu are compared in Supplementary Table 1. The 3D Cu possesses a much larger specific surface area than planar Cu and Cu foam. As a result of the high electroactive area ratio of 3D Cu (*r*=45), 98% of Li metal is deposited inside the 3D Cu structure. As shown in Fig. 6, for the planar current collectors, *r*=1 and *η*=0, indicating that all Li metal is deposited on surface of the electrode. For the Cu foam, *r*=5.2 and *η*=81%, indicating 19% of Li metal is deposited outside of the 3D structure. Although this formula is based on a simplified model, it explains why Li metal is accommodated in the 3D current collector rather than deposited at the top, even at higher current densities. Therefore, a high electroactive area ratio is of key importance for a 3D current collector accommodating Li metal and is a benchmark for effective 3D current collectors.

We have demonstrated that Li anode can be accommodated in the reserved pores of the 3D current collector with a submicron skeleton and high surface area, thereby suppressing the growth of dendritic Li and solving the associated problems of Li anodes. The accommodation of the Li anode into the reserved pores addresses the issue of dendrite growth remarkably. Hence, the Li metal anode can run for 600 h without resulting in short circuit, thereby significantly improving the cell life and safety of Li-metal batteries. Because of the submicron structure of the 3D current collector, the Li anode holds a high areal capacity and maintains a good plating/stripping efficiency of ∼98.5%. In addition, because of the high electroactive area of the submicron 3D structure, a high portion of Li metal is accommodated inside the 3D current collector and the voltage hysteresis and charge transfer resistance of the Li anode is reduced compared with that on a planar current collector. The porous current collector may not be limited to the porous Cu foil; other advanced porous architectures could also help improve the lifespan and performance of the Li anodes. It is noteworthy that combined means should be taken to develop the Li-metal anodes. The submicron-structured 3D current collector is expected to play a synergistic effect with other rationally designed cell components, including suitable electrolytes and additives, Li surface protective layers, modified Li metal, and so forth, to improve the comprehensive performance of the Li-metal anode. The utilization of the 3D architecture to accommodate the Li anode will facilitate further investigations on Li anodes and hasten the development of Li-metal batteries towards next-generation energy storage devices.

## Methods

### Synthesis

A Cu foil (25 μm in thickness, GoodFellow) was first washed by diluted hydrochloric acid and subsequently with deionized water to remove surface impurities. The Cu foil was immersed in an ammonia solution (5 wt%) for 36 h (or 48 h for thicker porous layer), during which the solution became blue and a cyan layer of Cu(OH)_2_ generated on the surface of the Cu foil. The foil with the cyan layer was washed by water and dried at 60 °C. It was finally heated at 180 °C for 4 h for dehydration and reduced at 400 °C for 10 h in a H_2_/Ar mixed flow (5% H_2_ in volume) to get the final 3D porous Cu foil. The 3D porous Cu was punched out into circular disks (*Φ*10 mm) as the 3D current collectors for Li anode after vacuum drying.

### Electrochemistry

CR2032-type coin cells were assembled to deposit Li on the current collectors to evaluate the Coulombic efficiency, electrochemical impedance spectra and other properties. The cells were assembled in an argon-filled glove box. The coin cell was composed of a Li foil as the counter/reference electrode, a Celgard separator, and a current collector as the working electrode. The electrolyte was 1 M LiTFSI in DOL/DME (1:1 by volume, 30 μl, BASF) without any additives unless noted otherwise. The Coulombic efficiency was tested at 0.5 mA cm^−2^ on a LAND electrochemical testing system at room temperature. The batteries were first cycled at 0–1 V (versus Li^+^/Li) at 50 μA for five cycles to stabilize the SEI and remove surface contaminations. After that, 1 mA h cm^−2^ of Li was deposited onto the current collector and then charged to 0.5 V (versus Li^+^/Li) to strip the Li at 0.5 mA cm^−2^ for each cycle. The Coulombic efficiency was calculated based on the ratio of Li stripping and plating. Electrochemical impedance spectra measurement was performed using an Autolab workstation (Metrohm) in the frequency range of 100 kHz to 100 mHz after specific cycles.

Symmetric cells were employed to evaluate the cycling stability and cycle life (short-circuit time *T*_sc_) of the Li anodes on different current collectors. The symmetric cell was assembled using a hollow spacer substituting for the Celgard separator in a CR2032-type coin cell, as illustrated in Supplementary Fig. 11. The electrolyte (1 M LiTFSI in DOL/DME, 200 μl) was carefully charged into the spacer without entrainment of bubbles. For the long-term galvanostatic discharge/charge test, 2 mA h cm^−2^ of Li was first deposited on the current collectors at 0.5 mA cm^−2^ and the cells were then charged and discharged at 0.2 mA cm^−2^ for 2.5 h in each half cycle. For the unidirectional galvanostatic polarization (accelerated test for *T*_sc_), Li was continuously plated onto the current collectors from the counter electrode at 0.5 mA cm^−2^ until short circuit. The average *T*_sc_ was obtained from at least three cells for each current collector.

For full cells with 3D Cu-based Li-metal anodes, LiFePO_4_ (Sanxin Industrial) was employed as cathode material. LiFePO_4_ was casted on an Al foil with an areal capacity density of ∼0.5 mA h cm^−2^. The 3D Cu was first assembled into a half cell using a Li foil as counter electrode. After plating 1 mA h cm^−2^ of Li metal into the 3D current collector, Li anode was extracted from the half cell and reassembled into a full cell against LiFePO_4_ cathode. The electrolyte was the same as that in the half cells (1 M LiTFSI in DOL/DME, 30 μl). Assembly of pouch cells was similar to that of the coin cells. The electrodes (∼42 cm^2^ in area) were stacked and assembled in a pouch cell. Li anodes plated in the 3D current collector were assembled into a pouch cell against LiFePO_4_ cathodes with 4 ml of the electrolyte to gain the pouch full cell with a capacity of ∼40 mA h.

### Characterization

The X-ray diffraction profiles of the as-obtained samples were obtained using an Empyrean X-ray diffractometer (PANalytical) with Cu Kα radiation (*λ*=1.54056 Å) operated at 40 kV and 40 mA. The top view and cross-sectional view images were observed on a field emission SEM (JEOL 6701F) and the 52° side view images of the Li anode were obtained from a focused ion beam microscope (Helios NanoLab 600i, FEI) with a tiltable specimen holder. An AFM system (Bruker Multimode 8 with a Nanoscope V controller) was employed to measure the height images of the anode surfaces. ToF-SIMS (TOF.SIMS5 IONTOF GmbH) was used to perform elemental analysis and depth profiles of the Li anode on the 3D Cu foil. A 20 keV Ar_n_^+^ (*n*=1,700) beam was used as sputter beam, which was scanned on an area of 300 × 300 μm^2^ at a sputter rate of ∼10 μm h^−1^. XPS was conducted on the ESCALab 250Xi (Thermo Scientific) using 200 W monochromatized Al Kα radiation. Porosity analysis was performed by a mercury porosimeter (AutoPore IV 9500, Micromeritics) with pressure from 0.1 to 60,000 psi.

For the *ex situ* analyses of the Li-metal anodes, batteries with specific discharge/charge states were first disassembled in the glove box to harvest the Li anodes. Before any characterization, the Li anodes were rinsed using DOL and DME solvents to remove residual electrolyte and LiTFSI salt and then dried in the glove box at ambient temperature. The Li anodes on planar Cu were not rinsed because the Li metal was loosely plated on the planar Cu collector and would be easily removed if washed. For *ex situ* SEM observations, the anodes were transferred through a specially designed device from the glove box to the vacuum chamber of the SEM without exposing them to air. For *ex situ* ToF-SIMS and XPS analyses, the samples were protected by argon and quickly transferred into the vacuum chamber. The samples were exposed to dry air for <30 s. The *ex situ* AFM images were scanned directly in argon atmosphere using an AFM apparatus mounted on top of a suspended marble in the glove box.

## Additional information

**How to cite this article:** Yang, C.-P. *et al.* Accommodating lithium into 3D current collectors with a submicron skeleton towards long-life lithium metal anodes. *Nat. Commun.* 6:8058 doi: 10.1038/ncomms9058 (2015).

## Supplementary Material

Supplementary Information Supplementary Figures 1-19 and Supplementary Table 1
