# Supplementary material for: Accommodating lithium into 3D current collectors with a submicron skeleton towards long-life lithium metal anodes
Source: Nat Commun. 2015 Aug 24;6:8058. doi: 10.1038/ncomms9058 (PMC4560781; doi:10.1038/ncomms9058)
Supplement: Supplementary Information — Supplementary Figures 1-19 and Supplementary Table 1 [file ncomms9058-s1.pdf]

Supplementary Figures

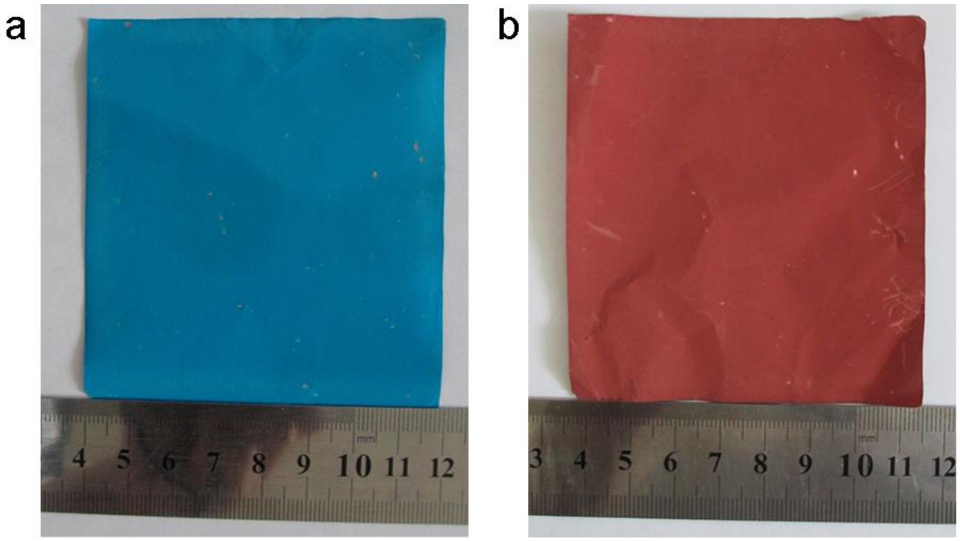

Supplementary Figure 1 | Photographs of the as-prepared samples. (**a**) $Cu(OH)_2$ on the Cu foil, (**b**) 3D Cu foil.

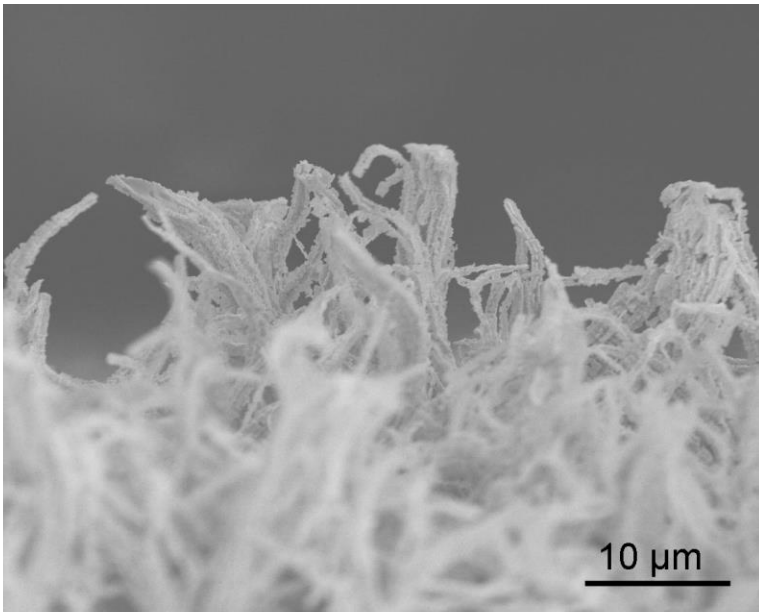

Supplementary Figure 2 | Side view SEM image of the porous Cu.

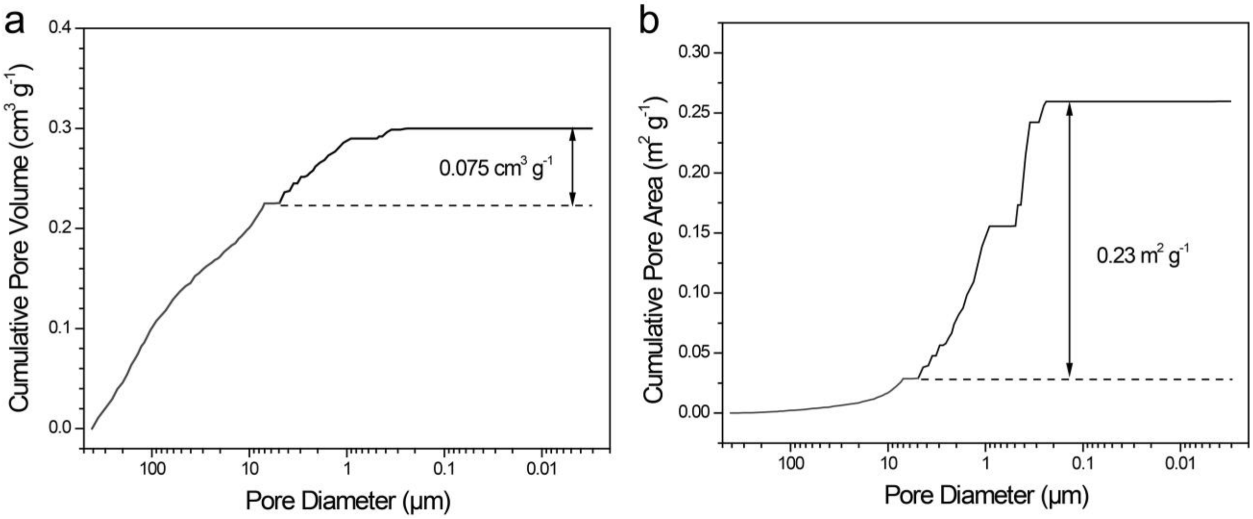

Supplementary Figure 3 | Porosity analysis for 3D Cu foil by mercury porosimetry. (**a**) Cumulative pore volume and (**b**) cumulative pore area of 3D porous Cu. Given that the pores of 3D Cu are generally smaller than 5 μm (see SEM image in Fig. 1c), pores larger than 5 μm are considered as interparticle voids during measurement. The effective pore volume of the 3D Cu foil is 0.075 cm^3 g^{-1}. The 3D Cu is averagely 19.4 mg cm^{-2}; thus, the effective pore volume of 3D Cu is 1.5×10^{-3} cm^3 cm^{-2}. The median pore diameter (by volume) of the 3D Cu foil is 2.1 μm. The specific surface area of 3D Cu is 0.23 m^2 g^{-1}, corresponding to a pore area per unit geometric area of 45 cm^2 cm^{-2}.

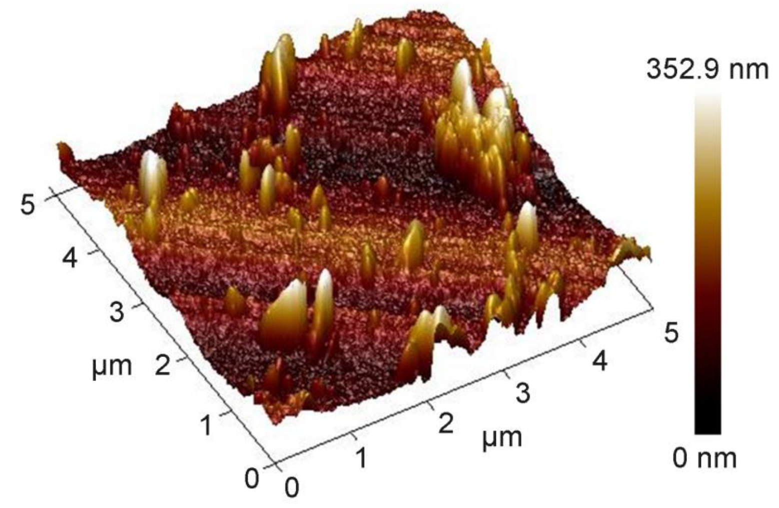

Supplementary Figure 4 | AFM image of Li plated on the planar Cu foil during the nucleation step.

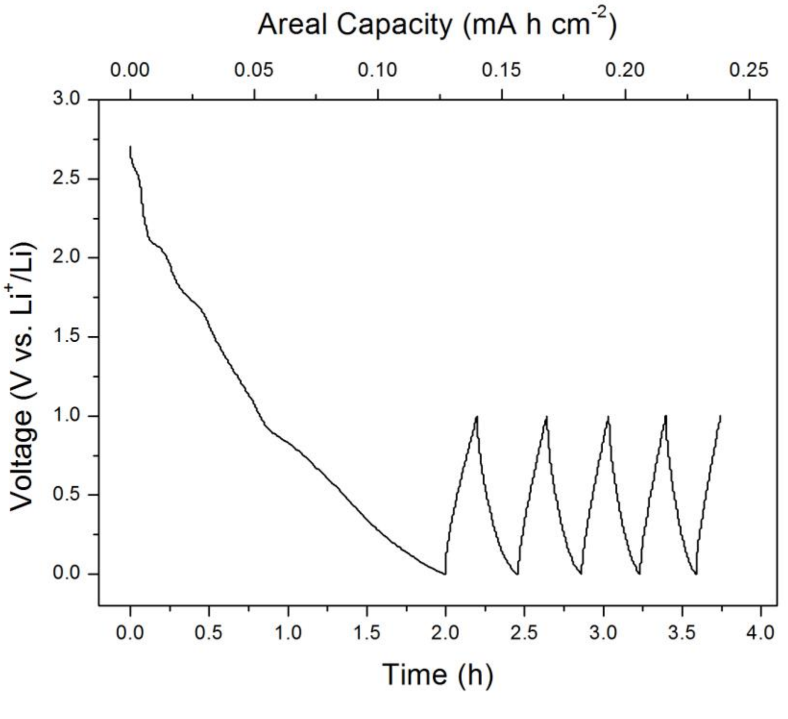

Supplementary Figure 5 | Typical voltage profile during initialization process. The batteries were first cycled at 0–1 V (vs. Li^+/Li) at 50 μA for five cycles for initialization prior to further electrochemical procedure.

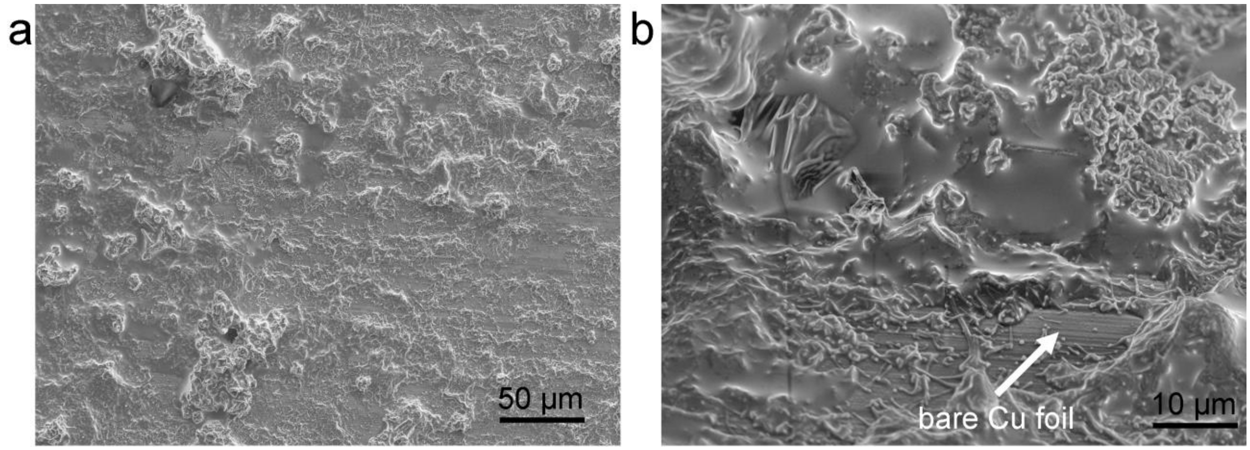

Supplementary Figure 6 | Morphology of Li metal plated on the planar Cu foil. (**a**) Top view and (**b**) side view SEM image of Li metal (2 mA h cm^{-2}) on the planar Cu.

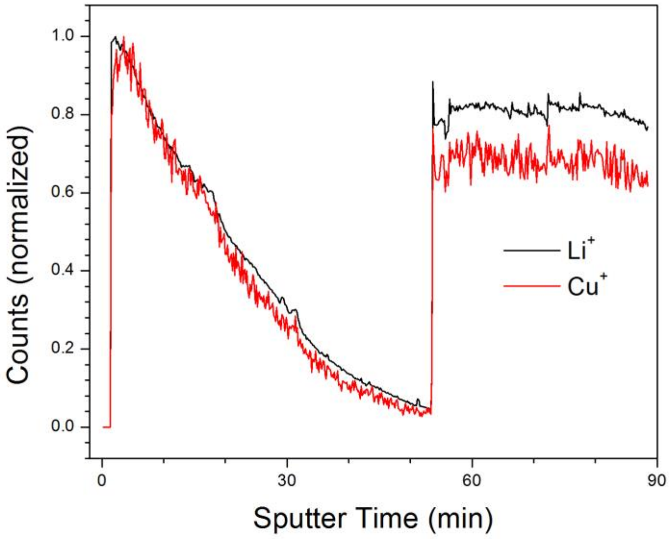

Supplementary Figure 7 | Depth distribution of Li and Cu elements by ToF-SIMS. Li^+ and Cu^+ profiles with sputter time (relevant to the sample depth) from the 3D porous Cu deposited with 2 mA h cm^{-2} of Li metal. The sputter rate was approximately 10 μm h^{-1}.

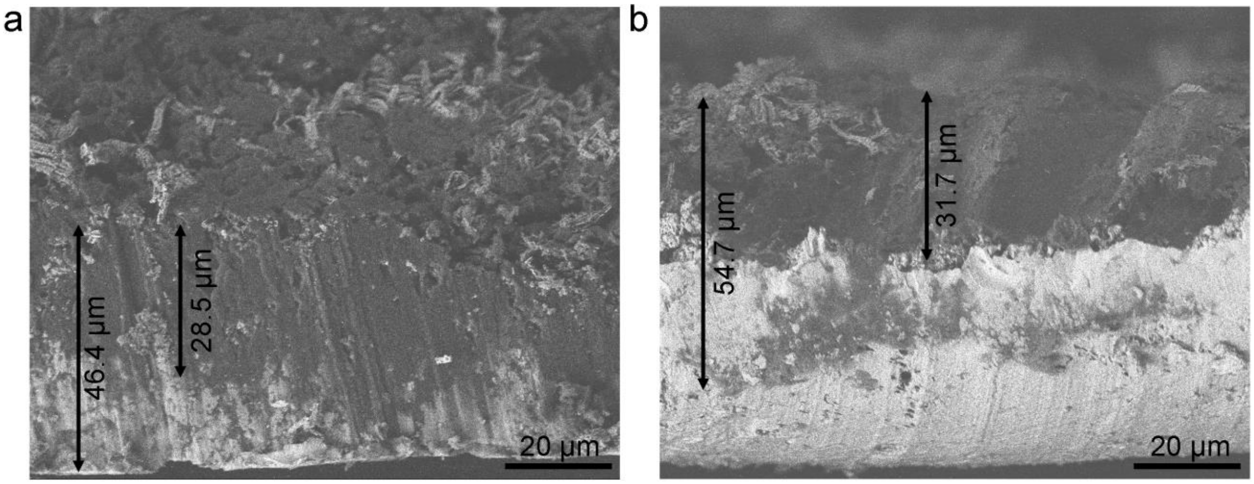

Supplementary Figure 8 | Morphologies of Li metal anodes on the 3D Cu foils at higher current densities. Cross sectional view SEM images of Li anodes (2 mA h cm^{-2}) deposited in 3D porous Cu at (**a**) 2 mA cm^{-2} and (**b**) 5 mA cm^{-2}.

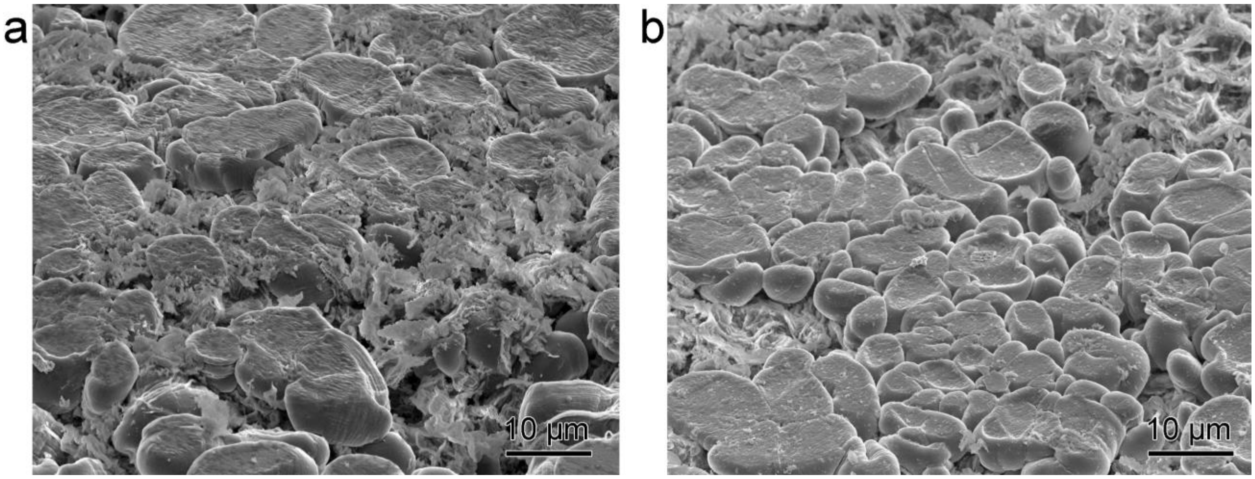

Supplementary Figure 9 | Morphologies of Li metal anodes on the 3D Cu foils after repeated cycles. Side view SEM images of Li anodes (2 mA h cm^{-2}) in 3D porous Cu after (**a**) 10 cycles and (**b**) 50 cycles.

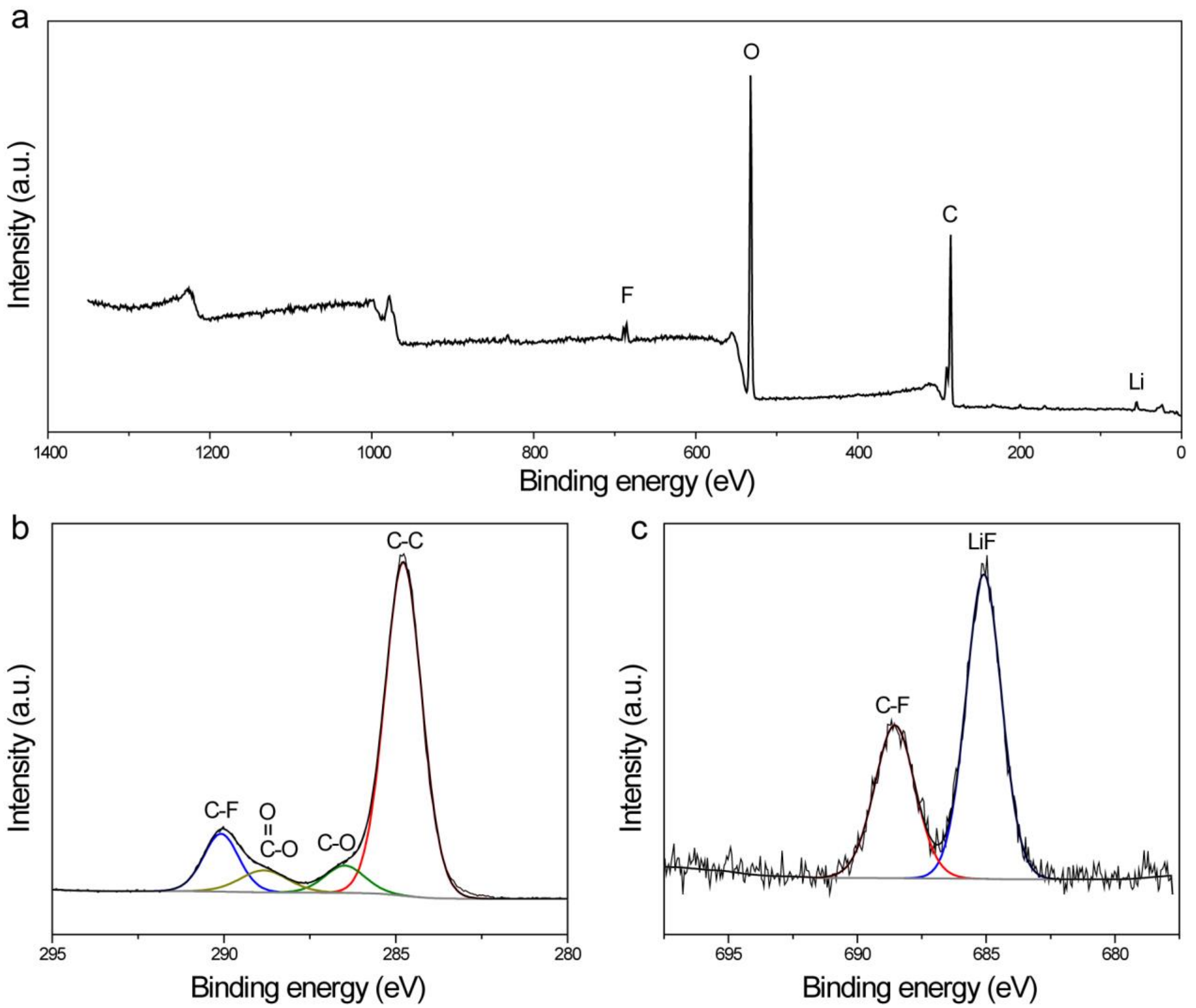

Supplementary Figure 10 | XPS spectra of Li metal anode on the 3D Cu foil after 10 cycles. (**a**) Survey, (**b**) C 1s, and (**c**) F 1s spectra of the plated Li metal anode (2 mA h cm^{-2}). The fitted profiles of C 1s and F 1s spectra shows the major groups of the SEI layer are C-C, C-O, COO, C-F, LiF, et al. Considering the electrolyte constitution, the major components of the SEI film of the Li metal anode on the 3D Cu foil are ROLi, ROCOOLi, LiF, et al.

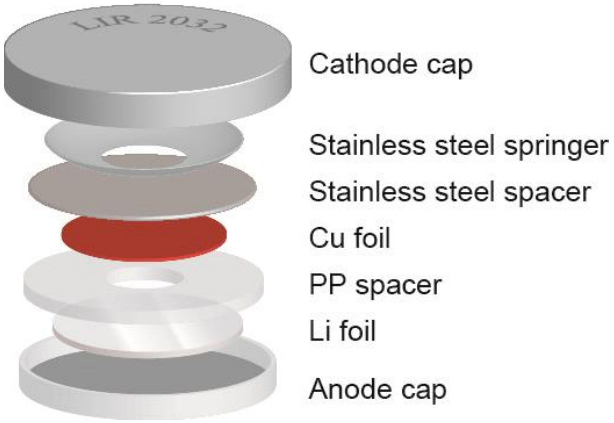

Supplementary Figure 11 | Schematic presentation of a symmetric Li cell. The symmetric Li cell uses a Li foil as the counter/reference electrode, a Cu foil (planar foil or 3D porous foil, or replaced with Li foil) as the working electrode, and a hollow propene polymer (PP) spacer substituting for the separator to allow the growth of dendritic Li. The inner diameter of the PP spacer is 6 mm and the thickness is 1 mm.

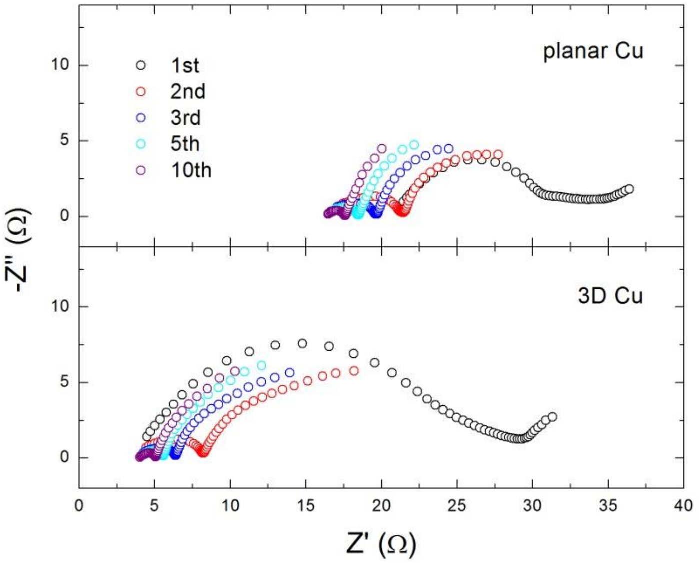

Supplementary Figure 12 | Comparison of resistance of Li anodes on planar and 3D Cu current collectors during cycling. EIS spectra of Li plated on planar Cu foil and 3D Cu foil after 1st, 2nd, 3rd, 5th, and 10th Li-plating.

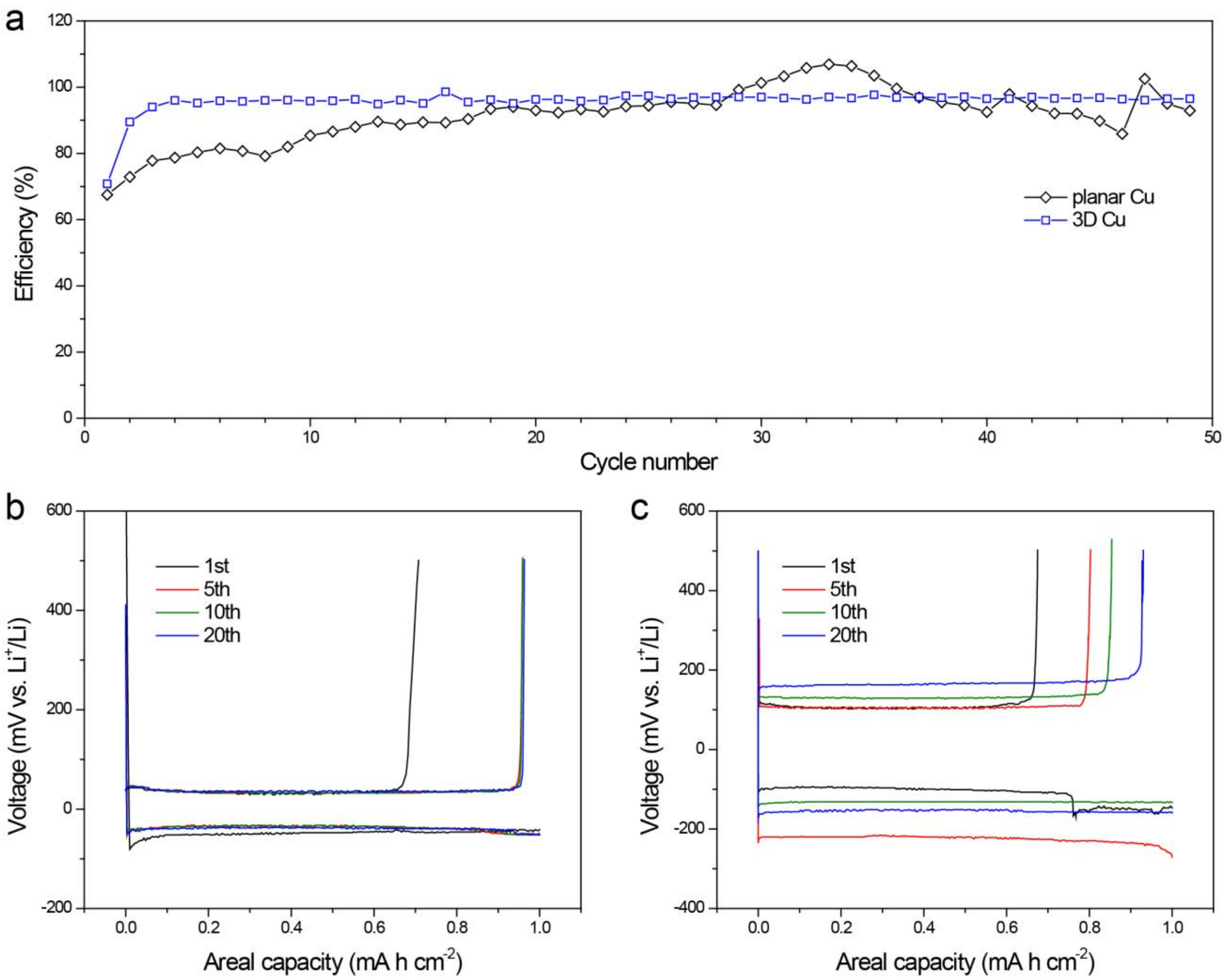

Supplementary Figure 13 | Coulombic efficiencies of Li metal anodes with different current collectors. (**a**) Coulombic efficiencies of Li anodes on planar Cu and 3D Cu. Discharge/charge voltage profiles of Li plating/stripping on (**b**) 3D Cu and (**c**) planar Cu. Discharge/charge were conducted at 0.5 mA cm^{-2}. Prior to the efficiency test, the batteries were first cycled at 0–1 V at 50 μA for five cycles for contamination removal and interface stabilization.

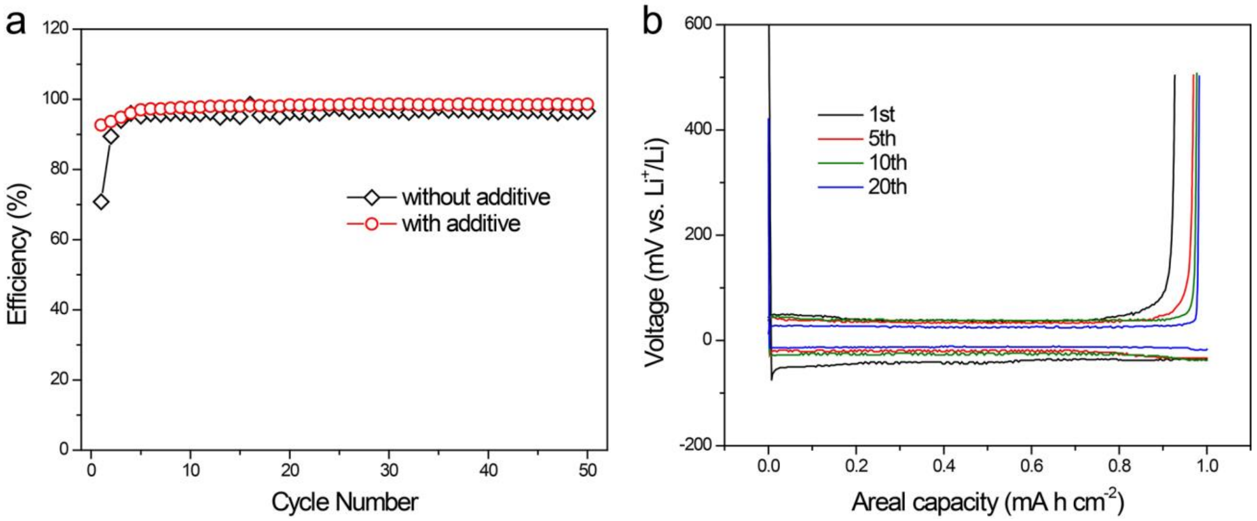

Supplementary Figure 14 | Improving Coulombic efficiencies of Li metal anode in 3D Cu by employing an electrolyte additive. (**a**) Coulombic efficiencies of Li metal anode in 3D Cu current collectors with or without electrolyte additive. The electrolyte additive is 1% $LiNO_3$ and 0.005 M Li_2S_6. The additive generally exists in Li–S batteries. (**b**) Discharge/charge voltage profiles of Li plating/stripping with the electrolyte additive. Discharge/charge were conducted at 0.5 mA cm^{-2}. Prior to the efficiency test, the batteries were first cycled at 0–1 V at 50 μA for five cycles for contamination removal and interface stabilization. Compared with the battery without any additives, the one with 1% $LiNO_3$ and 0.005 M Li_2S_6 shows significantly improved initial Coulombic efficiency (from 71% to 93%) and has a stable efficiency of ~98.5%.

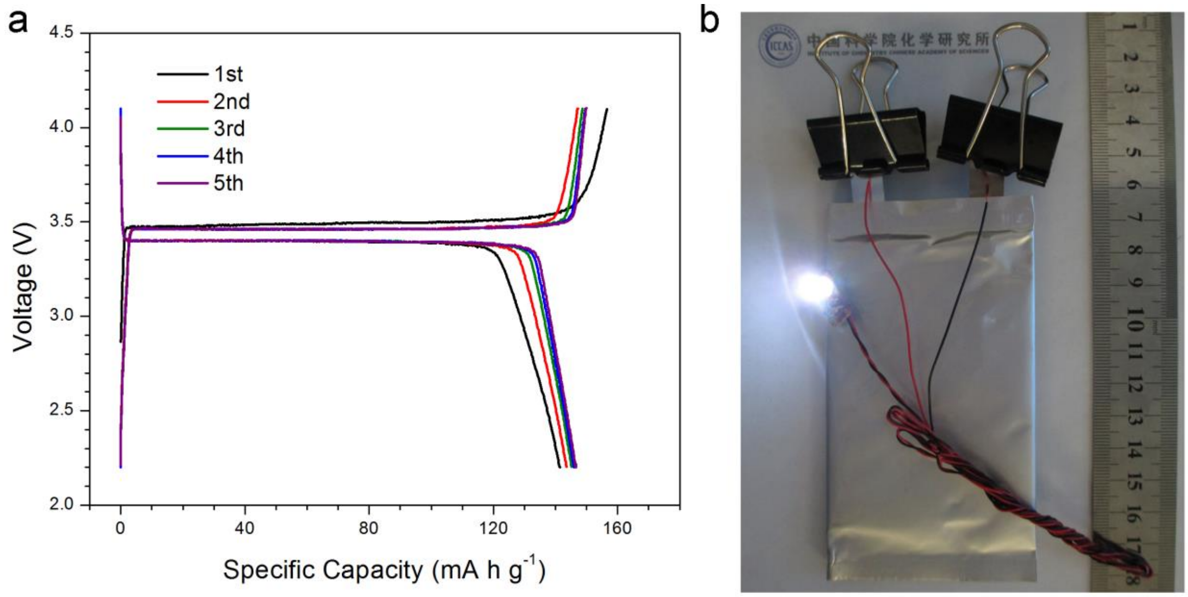

Supplementary Figure 15 | Full-cells using 3D Cu based Li anode and $LiFePO_4$ cathode. (**a**) Electrochemical performance of the full cell run at 0.2 C (based on the $LiFePO_4$ cathode, ~0.1 mA cm^{-1}). The capacity increased during the first several cycles could be attributed to an activation process of the $LiFePO_4$ cathode used. (**b**) Pouch cell of 3D Cu based Li anode and $LiFePO_4$ cathode. The pouch cell has a capacity of ~40 mA h. The photo here shows the charged pouch cell powering an LED device.

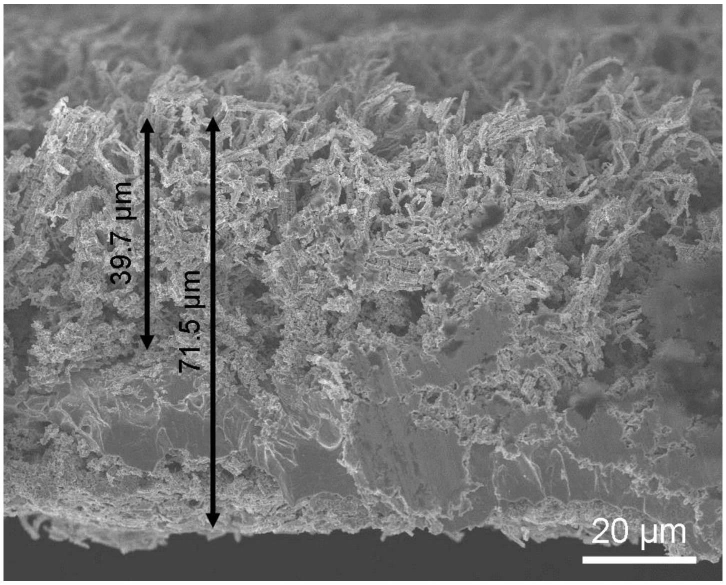

Supplementary Figure 16 | 3D Cu foil with more abundant pores. Cross sectional SEM image of a 3D Cu foil that was immersed in the ammonia solution for 2 days and has a larger pore volume.

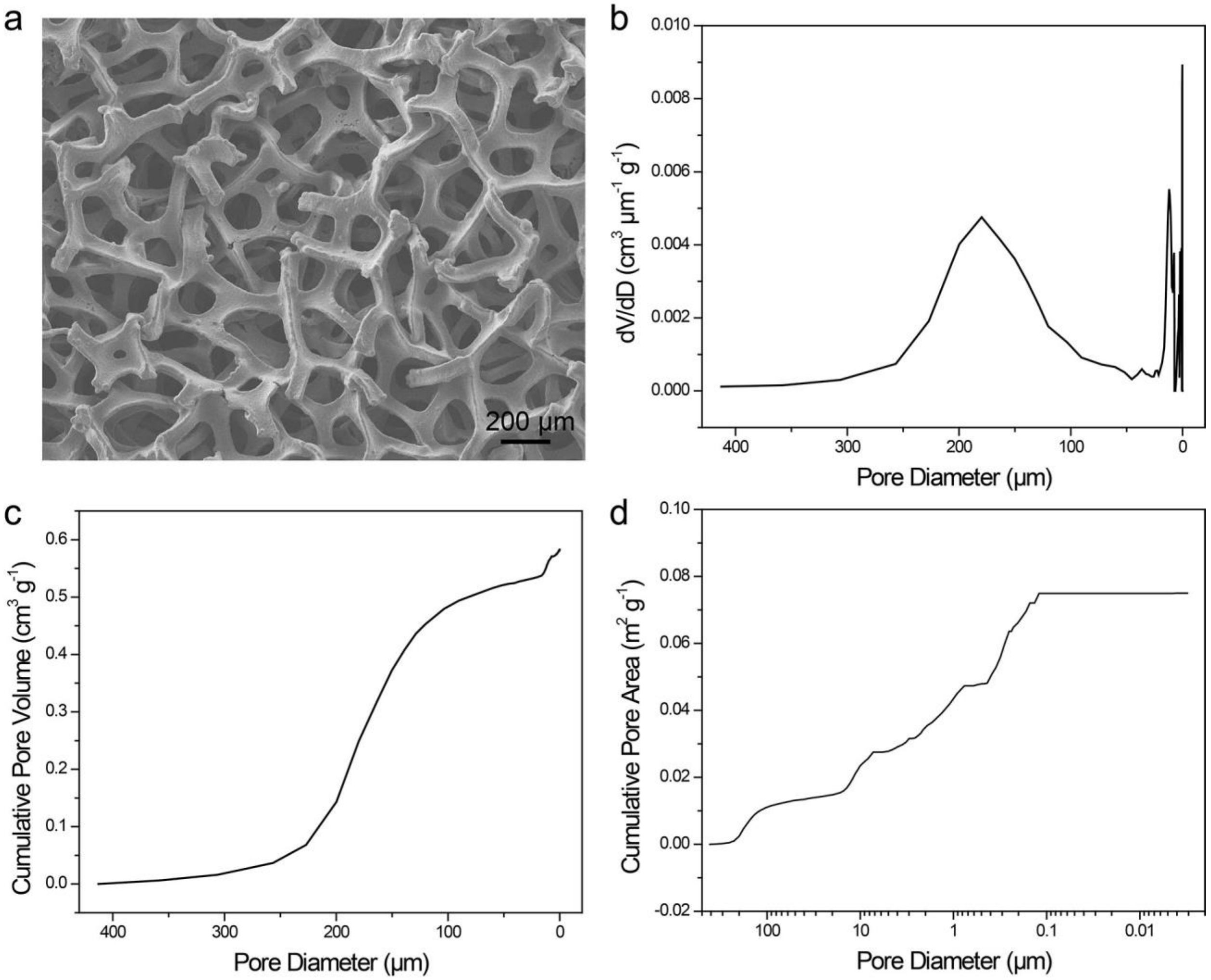

Supplementary Figure 17 | Morphology and porosity analysis of Cu foam by mercury porosimetry. (**a**) SEM image, (**b**) pore diameter distribution, (**c**) cumulative pore volume, and (**d**) cumulative pore area of Cu foam. The median pore diameter (by volume) of Cu foam is 170 μm. The Cu foam is averagely 6.93 mg cm^{-2}. The total intrusion volume is 0.58 cm^3 g^{-1}, corresponding to an areal pore volume of 4.0×10^{-3} cm^3 cm^{-2}. The specific surface area of Cu foam is 0.075 m^2 g^{-1}, corresponding to a pore area per unit geometric area of 5.2 cm^2 cm^{-2}.

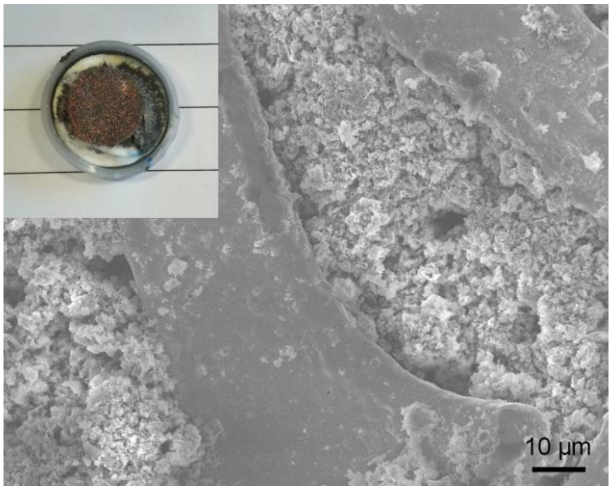

Supplementary Figure 18 | Li deposition in Cu foam. SEM image and digital photo (inset) of Li anode with Cu foam current collector after 20th Li-stripping.

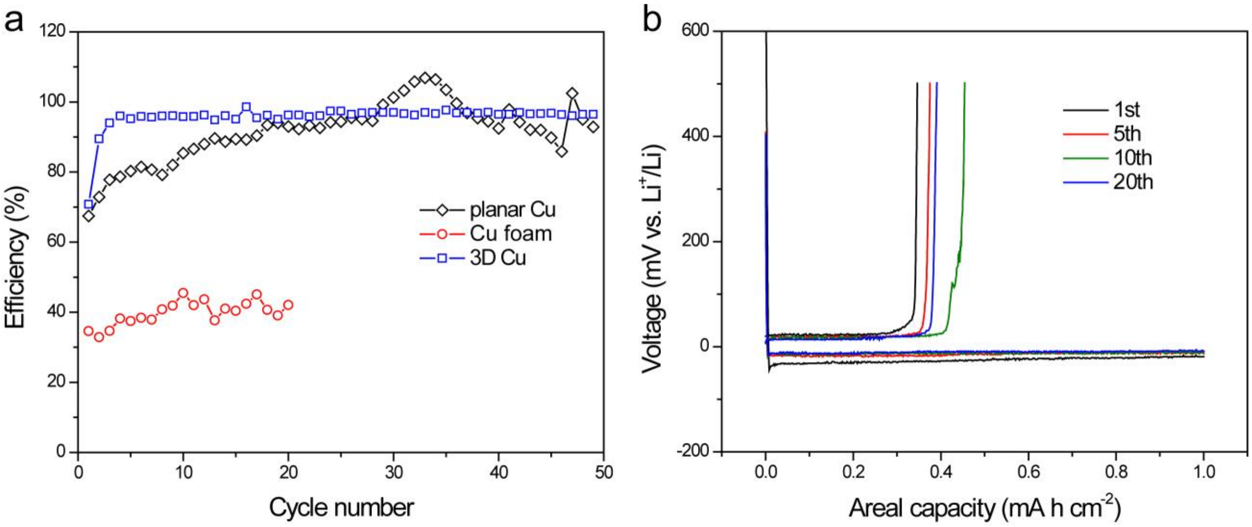

Supplementary Figure 19 | Coulombic efficiency of Li metal anode with Cu foam current collectors. (**a**) Coulombic efficiency of Li plating/stripping in Cu foam in comparison with that on planar Cu and Cu foam. (**b**) Discharge/charge voltage profiles of Li plating/stripping in Cu foam. Discharge/charge were conducted at 0.5 mA cm^{-2}. Prior to the efficiency test, the batteries were first cycled at 0–1 V at 50 μA for five cycles for contamination removal and interface stabilization.

Supplementary Table

Supplementary Table 1 | Textural parameters of different current collectors.

	planar Cu	Cu foam	3D Cu*
Areal density (mg cm^{-2})	21.8	6.93	19.4
Median pore diameter (by volume, μm)	–	170	2.1
Specific pore volume (cm^3 g^{-1})	–	0.58	0.075
Areal pore volume (10^{-3} cm^3 cm^{-2})	–	4.0	1.5
Specific surface area (m^2 g^{-1})	–	0.075	0.23
Electroactive area ratio**	1	5.2	45

* Median pore diameter, pore volume, and pore area are based on effective pores (pore diameter < 5 μm).

** Ratio of the electroactive surface area exposed to the electrolyte to the geometric area of the current collector. Electroactive area ratio = specific surface area × areal density.
